# Optimization of glutaminase-free L-asparaginase production using mangrove endophytic *Lysinibacillus fusiformis* B27

**DOI:** 10.12688/f1000research.21178.2

**Published:** 2020-05-14

**Authors:** Asep Awaludin Prihanto, Indah Yanti, Mohammad Achsanil Murtazam, Yoga Dwi Jatmiko

**Affiliations:** 1 Department Fishery Product Technology, Faculty of Fisheries and Marine Science, Brawijaya University, Malang, East Java, 65145, Indonesia; 2 BIO-SEAFOOD Research Unit, Faculty of Fisheries and Marine Science, Malang, East Java, 65145, Indonesia; 3 Department of Mathematic, Faculty of Natural Science and Mathematic, Brawijaya University, Malang, East Java, 65145, Indonesia; 4 Department of Biology, Faculty of Natural Science and Mathematic, Brawijaya University, Malang, East Java, 65145, Indonesia

**Keywords:** L-asparaginase, mutation, statistical, taguchi, Lysinibacillus fusiformis.

## Abstract

**Background: **The mangrove, *Rhizophora mucronata*, an essential source of endophytic bacteria, was investigated for its ability to produce glutaminase-free L-asparaginase. The study aimed to obtain glutaminase-free L-asparaginase-producing endophytic bacteria from the mangrove and to optimize enzyme production.

**Methods: **The screening of L-asparaginase-producing bacteria used modified M9 medium. The potential producer was further analyzed with respect to its species using 16S rRNA gene sequencing. Taguchi experimental design was applied to optimize the enzyme production. Four factors (L-asparagine concentration, pH, temperature, and inoculum concentration) were selected at four levels.

**Results:** The results indicated that the endophytic bacteria *Lysinibacillus fusiformis* B27 isolated from *R. mucronata* was a potential producer of glutaminase-free L-asparaginase. The experiment indicated that pH 6, temperature at 35°C, and inoculum concentration of 1.5% enabled the best production and were essential factors. L-asparagine (2%) was less critical for optimum production.

Conclusions: L. fusiformis B27, isolated from *Rhizophora mucronata*, can be optimized for L-ASNase enzyme production using optimization factors (L-ASNase, pH, temperature, and inoculum), which can increase L-ASNase enzyme production by approximately three-fold.

## Introduction

L-asparaginase (L-ASNase, EC.3.5.1.1, L-asparagine amidohydrolase) hydrolyzes L-asparagine (L-ASN) to L-aspartic acid and ammonia ^1^. This enzyme has an essential role in food safety owing to its acrylamide-mitigating potential ^2, 3^. Acrylamide, a compound formed from the reaction of asparagine with reducing sugars, can be converted into L-asparagine (ASN) in raw materials by adding L-ASNase. The food industry mainly uses two commercial products, Acrylaway and Preventase from *Aspergillus oryzae* and *Aspergillus niger*, respectively ^4, 5^.

L-ASNase can also inhibit the growth of cancer cells, especially leukemia ^4^, and it is potentially suitable for leukemia treatment due to its ability to deplete L-ASN in blood plasma ^5^. Cells in healthy tissue can synthesize L-ASN in sufficient amounts for protein synthesis, but some types of lymphoid malignancies remove it from plasma. Limiting the amount of L-ASN will eventually inhibit the growth of cancer cells. Applying L-ASNase to the treatment of acute lymphoblastic leukemia, acute myelocytic leukemia, Hodgkin’s disease, non- Hodgkin’s disease, and melanosarcoma has been investigated for its effectiveness in animals and humans ^6^.

Some characteristics of this enzyme hinder its application in foods and medicines. For example, it is unsuitable for application in foods due to its sensitivity to high temperatures ^6^. In medical applications, it has a low half-life and can cause allergic reactions in patients ^6^. Some of these operational and technical constraints cause manufacturers to identify new sources of L-ASNase. Therefore, new L-ASNase producers need to be explored.

L-ASNase is commonly found in animal tissues, bacteria, and plants, but is lacking in humans. L-ASNase is produced in large quantities by several microorganisms such as *Enterobacter aerogenes, Escherichia coli, Erwinia carotovora, Enterobacter aerogenes, Candida sp.,* and *Corynebacterium glutamicum* ^7, 8^. Production of L-ASNase sourced from animals or plants encounter several impediments, including those relating to large-scale enzyme production. Therefore, the production of L-ASNase derived from microorganisms is preferred because these multiply rapidly and are easily managed ^9^.

Optimizing the growth parameters improves enzyme production. Enzyme production can be optimized via a statistical approach using Taguchi analysis. The Taguchi method facilitates product or process design ^10^. This approach offers time efficiency and accurate results, using relatively few treatment parameters ^11^.

Considering the drawbacks of the L-ASNase toward medical and food application, and for fulfilling the need for L-ASNase with better properties, The search for exploring the new source of L-ASNase is remarkably important. This study was conducted to isolate and identify potential endophytic bacteria producing the L-ASNase enzyme from the mangrove *Rhizophora mucronata*. Optimization of L-ASNase enzyme production by selected bacteria was performed by optimizing factors influencing production (L-ASN, pH, temperature, inoculum concentration, and incubation time) using a statistical Taguchi approach.

## Methods

### Isolation and screening of L-ASNase producers

Isolation and screening of L-ASNase-producing endophytic bacteria was performed as described by Prihanto *et al.* ^12^ and Mahajan *et al*. ^13^. Mangrove *(Rhizophora mucronata)* was obtained from Aeng Sareh Beach, Madura Island, Indonesia with the location of 7°13'5.57"S and 113°19'8.89"E. Mangrove stem was aseptically cut to approximately 1 cm diameter. The sample was placed in a plastic bag and immediately transported to the laboratory on ice (4°C). It was crushed and weighed (1 g) and then serially diluted with physiological buffer. Three independent treatments were applied. An appropriately diluted sample (10 ^–3^–10 ^–5^) was plated onto Luria-Bertani agar (Sigma-Aldrich, USA) using the pour plate method and incubated for 72 h at 35°C. Colonies were purified in Luria-Bertani agar (Sigma-Aldrich, USA) using three quadrants streaking, and stored at −20°C.

A modified M9 medium was used for screening isolates that were produced glutaminase-free L-ASNase. All materials and reagents were purchased from Merck, USA. The composition of the medium was 6 g/l of Na _2_HPO _4_, 2 g/l of KH _2_PO _4_, 0.5 g/l of NaCl, 20 g/l of L-ASN or L-glutamine, 2 g/l of glucose, 0.2 g/l of MgSO _4_, 0.005 g/l of CaCl _2_, agar 2%, and Bromotymol blue (BTB) 0.007%. The pH of the medium was set to 5.5 using a pH meter with 2 N HCl. BTB served as a color indicator. Two media (M9 medium with L-ASN, and M9 medium with L-glutamine) were applied to the isolates. Glutaminase-free L-ASNase isolates were detected by the ability to hydrolyze only L-ASN.For isolate which able to hydrolyze both L-ASN, and L-glutamine was not chosen for further analysis. Initial identification of the selected isolates was performed by Gram staining.

### Identification of bacterial isolates

Molecular identification of the selected bacterial isolates from mangrove was performed based on the methods of Prihanto *et al.* ^12^. Genomic DNA from bacteria was amplified using 27F primers (5’-AGAGTTTGATCATGGCTCAG-3') and 1492R (5'-TACGGCTACCTTGTTACGA-3'). Primers were purchased from 1 ^st^ BASE (Singapore). Genomic DNA of bacteria were extracted, and amplified by following the company manual procedures (Wizard ^TM ^DNA purification Kit, Cat. No. A1120, and Gotaq® DNA polymerase, Cat No. M3005, Promega, USA). Obtained DNA sample (1 μL) was mixed with 18.5 μL double distilled H _2_O (ddH _2_O), 2.5 μL Buffer B with Mg2 + 10X, 1 μL dNTPs, 1 μL forward primer, 1 μL reverse primer, and 0.2 μL Taq polymerase. The reaction was performed in a thermocycler (T100 Thermal Cycler, Bio-Rad, USA) (denaturation: 94°C, 45 s; annealing: 61°C, 45 s; elongation: 72°C, 2 min). The amplification process was performed over 32 cycles. The Polymerase Chain Reaction results were checked by gel electrophoresis (Mupid EXu submarine, Takara, Japan) under 80 volt and 40 mA with 1.5% agarose. The gel was visualized using Benchtop UV Transilluminator (UVP, Canada).

The amplicon was further sequenced with ABI PRISM 3130×1 DNA sequencer (Applied Biosystems, USA). The sequence homology was investigated at the National Center for Biotechnology Information (NCBI) GenBank database using BLAST nucleotide programs, on the nucleotides collection database by excluding human and mouse genomic. The program was optimized for highly similar sequences (megablast). A phylogenetic tree was constructed using the UPGMA method with the MAFFT online ver 7. phylogenetic analysis service ^14^.

### Optimization of L-ASNase production

All materials and reagents were purchased from Merck, USA. Enzyme production was performed in a medium containing trisodium citrate 0.375 g, (NH _4_) _2_HPO _4_ 0.1 g, K _2_HPO _4_ 0.00625 g, MgSO _4_·7H _2_O 0.01 g, FeSO _4_·7H _2_O 0.001 g, yeast extract 0.075 g, and L-ASN 0.25–1 g ^15^. Different volume of inoculum (10 ^8^ CFU/ml) was inoculated on 50 ml fresh treated-production mediums. The cultures were incubated in a shaker incubator (120 rpm) at different temperatures. Four factors at four levels, L-ASN (0.5–2%), pH (6–9), temperature (30–45°C), and inoculum volume (0.5–2%), were investigated ( Table 1). The L16 orthogonal array was selected for experimental design ( Table 2). After 24 h incubation, the culture was harvested and the production of enzyme was investigated.

**Table 1.** Selected factors and levels for L-asparaginase production.

Factors	Level			
	1	2	3	4
**L-asparagine**	0.5%	1%	1.5%	2%
**pH**	6	7	8	9
**Temperature**	30°C	35°C	40°C	45°C
**Inoculum**	0.5%	1%	1.5%	2%

**Table 2.** Orthogonal array of designed experiment (L16).

Exp. No.	Factor level				L-ASNase activity (U/ml)
	L-ASN	pH	Temp	Inoculum	
**1**	1	1	1	1	3.32±0.03
**2**	1	2	2	2	15.43±0.25
**3**	1	3	3	3	0.42±0.08
**4**	1	4	4	4	12.02±1.30
**5**	2	1	2	3	11.56±2.08
**6**	2	2	1	4	3.68±0.01
**7**	2	3	4	1	10.83±0.57
**8**	2	4	3	2	5.02±0.06
**9**	3	1	3	4	6.47±1.57
**10**	3	2	4	3	9.11±0.33
**11**	3	3	1	2	6.74±0.08
**12**	3	4	2	1	11.45±0.91
**13**	4	1	4	2	7.09±0.08
**14**	4	2	3	1	1.09±0.04
**15**	4	3	2	4	16.04±1.38
**16**	4	4	1	3	4.15±0.34

### L-ASNase assay

A slightly modified method ^16^ was used for the enzyme activity assay. The samples (150 μl) were transferred to microtubes to which 100 μl of 150 mM L-ASN, 200 μl aquades, and 50 μl of 1 M phosphate buffer (pH 7) were added before homogenization. The samples were then incubated in a water bath at 30°C for 30 min to react. Subsequently, 100 μl of 1.5 M Trichloro Acetic Acid was added to stop the reaction followed by centrifugation at 2,000 rpm for 12 min; the supernatant (450 μl) was then transferred into the microtube. Nessler’s reagent (125 μl) was then added and reacted for 15 min until the color changed; the absorbance was read with a UV–vis spectrophotometer at a wavelength of 480 nm. NH _4_Cl served as standard ammonia. The enzyme activity was expressed as micromoles ammonia released per minute.

The activity was calculated with the following formula:

Enzyme activity (U/ml) $=\;\frac{\left(\mu \mathrm{mol}\;\mathrm{of}\;\mathrm{liberated}\;NH3)x\;(0.6\right)}{(0\text{.45)×}\;\text{(30)}\;\text{×}\;(0\text{.1)}}\;$

Where 0.6 = initial volume of enzyme mixture (ml)

            0.45 = volume of enzyme mixture used in final reaction (ml)

            30   = incubation time (min)

            0.1   = volume of enzyme used (ml)

### Data analysis

All L16 data were analyzed using Qualitek-4 software (Nutek Inc., MI). The analysis was used to obtain the predicted optimum conditions for achieving the highest L-ASNase production. Analysis of variance (ANOVA) was used to identify factors that influenced L-ASNase production. The F-ratio was calculated with a 95% confidence interval. Interactions among factors and levels of experiment was analyzed by determining the severity index (SI)

Experimental validation was conducted to confirm the accuracy of the obtained results. This aimed to verify the optimum factors and levels obtained from the experiment. After determining the optimum treatment, the next step was to conduct a confirmatory experiment by creating the optimum conditions based on the predetermined factors and levels.

## Results and discussion

### Screening and identification

**Figure 1.**
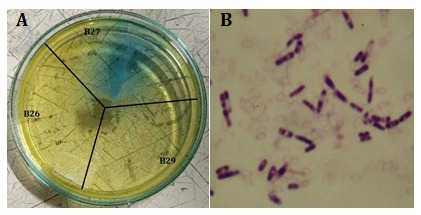
Results of L-asparaginase screening producing endophytic bacteria B27 isolated from *Rhizophora mucronata*. ( **A**) Blue color indicating the production of glutaminase-free L-asparaginase. ( **B**). Gram stain of B27 isolate.

The screening results showed that 31 endophytic mangrove isolates could produce the L-ASNase enzyme. Judging from the blue color of the medium, isolate B27 was the best producer ( Figure 1A). Isolate B27 had a more extensive and darker blue tone compared to other isolates. Furthermore, only isolate B27 produced glutaminase-L-asparaginase. Hence, further molecular identification was applied only to the B27 isolate. Further, Gram stain analysis revealed that the bacteria was Gram positive ( Figure 1B). 

**Figure 2.**
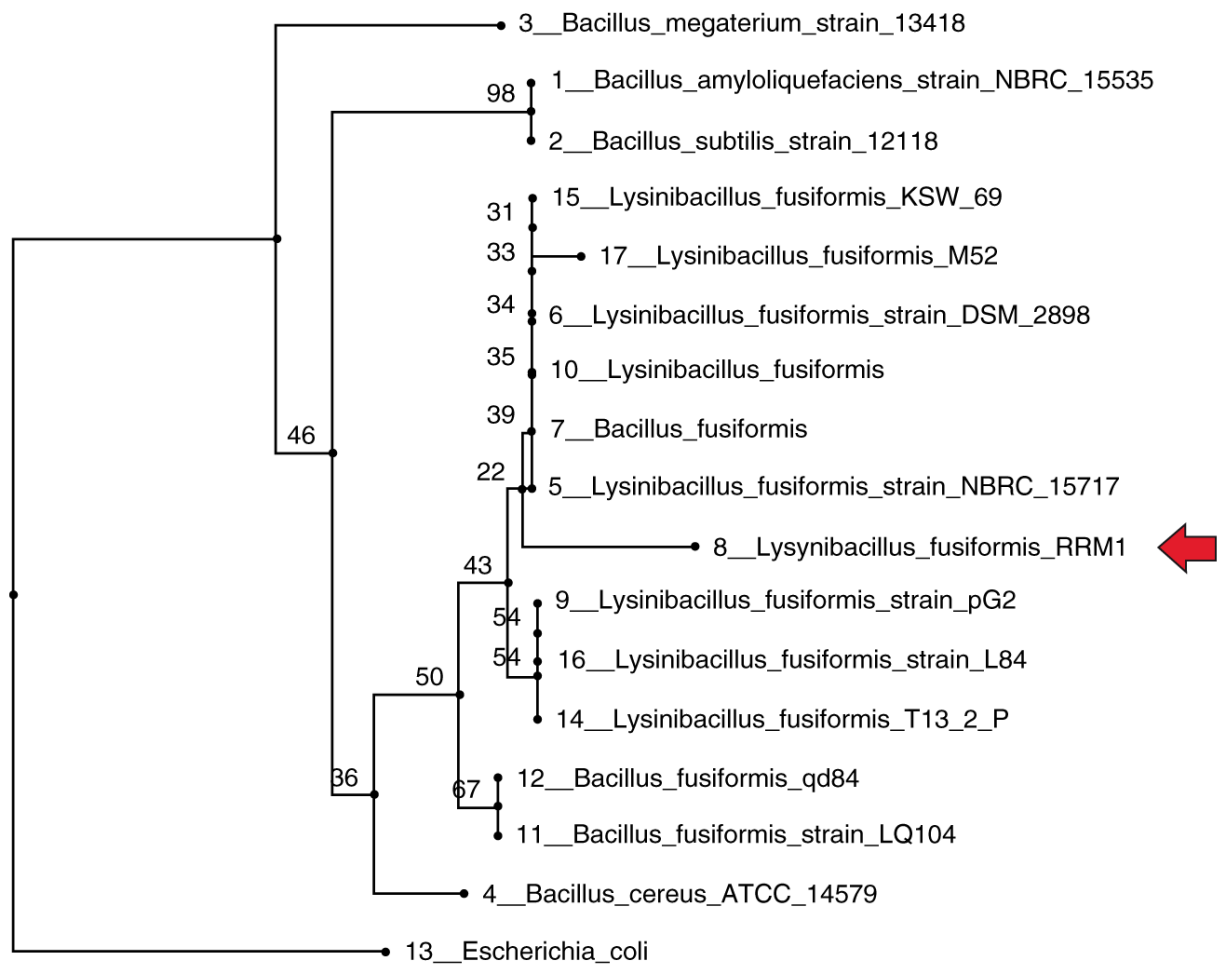
UPGMA phylogenetic tree of *Lysinibacillus fusiformis* B27 isolated from *Rhizophora mucronata*.

The 16S rDNA molecular identification results showed that B27 isolates had a similarity of 98% with the *Lysinibacillus fusiformis* species. The phylogenetic analysis results showed that *L. fusiformis* was most closely related to *L. fusiformis* strain NBRC15717 ( Figure 2). The production of L-ASNase from this bacteria was further optimized using the Taguchi method.

### Optimization of L-ASNase production

L-ASNase production was controlled to determine enzyme production without optimization. The enzyme production during incubation periods of 24 and 48 h was 3.18 and 1.99 U/ml, respectively. The higher production was observed after 24 h. During the exponential phase, bacteria experience fast growth and produce metabolites for growth and self-defense ^17^. The result for optimization experiment is shown in Table 2.

### Influence of culture factors

**Figure 3.**
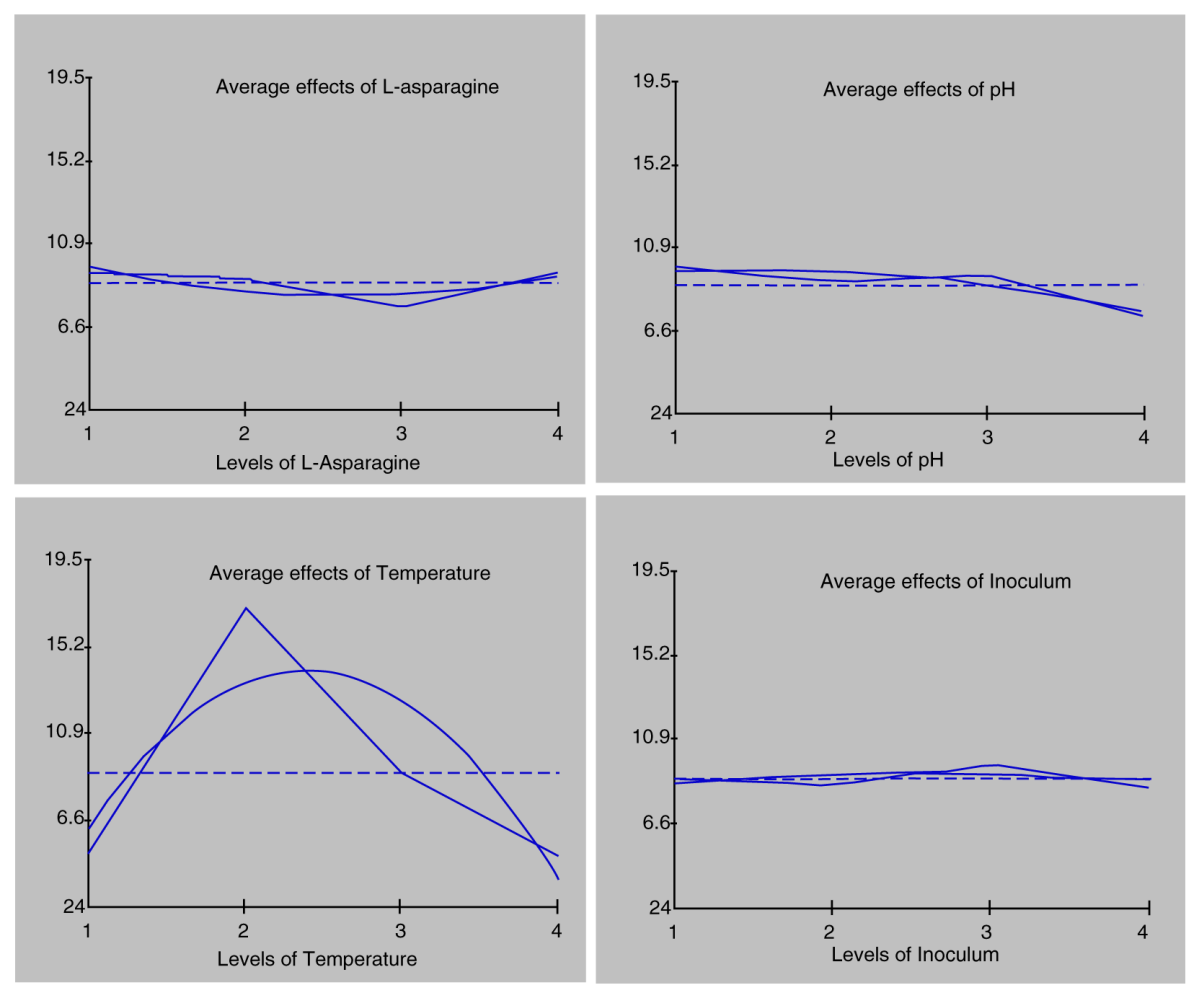
Impact of selected variables (L-asparagine, pH, temperature and inoculum) on the production of L-asparaginase producing bacteria isolated from *Rhizophora mucronata.* Angled lines is the enzyme activity. Non-angled lines is regression function of the enzyme activity.

To determine the effects of factors that could increase the L-ASNase production, L-ASN concentration (0.5, 1, 1.5, and 2%), pH (6, 7, 8, and 9), temperature (30, 35, 40, and 45°C), and inoculum concentration (0.5, 1, 1.5, and 2%) were evaluated. L-ASN concentration was used to determine the effect of inducers on increasing enzyme production. pH, temperature, and inoculum concentration were subsequently investigated. The effects of these factors are shown in Figure 3.

L-ASN concentration influenced L-ASNase production, with the best concentration being 2% L-ASN with enzyme production of 9.466 U/ml. Therefore, to some extent, L-ASN could increase enzyme activity. However, the results showed that L-ASN concentration 1% could reduce enzyme production. Consequently, the higher the substrate concentration added to the enzyme solution, the lower the enzyme activity until the enzyme reached the optimum substrate concentration ^18^. This data suggested that the enzyme production exhibited an optimum concentration of more than 2% L-ASN in the medium.

*L. fusiformis* B27 at pH 6 showed an enzyme production of 9.795 U/ml. When the pH was too high or low, enzyme production was reduced. Therefore, the optimum pH for enzyme production must always be considered. *L. fusiformis* B27 grown at 35°C showed the highest enzyme production of 17.037 U/ml. Temperature plays a vital role in enzymatic reactions. High temperatures increase the reaction rate of the enzyme. Hence, the optimum temperature for enzymes needs to be determined.

The optimum inoculum concentration for L-ASNase production was 1.5% with an enzyme production of 9.638 U/ml. A very high inoculum concentration can cause competition between microbes in obtaining nutrition. This competition can cause some microbes to lack the nutrients required for growth ^19, 20^.

### Factors influencing enzyme production

Analysis of variance (ANOVA) was used to identify factors that influenced L-ASNase production ( Table 3). The F-ratio was calculated with a 95% confidence interval. The results showed that all factors significantly influenced enzyme production. The most influential factor affecting L-ASNase enzyme production was temperature, while the inoculum concentration, although it was known to affect the enzyme production, showed the least effect.

**Table 3.** ANOVA analysis for investigated factors.

Factor	DOF (f)	Sum of squares	Variance (V)	F-Ratio (F)	Pure sum (S')	Percent P (%)
L-asparagine	3	16.195	5.,398	2.633	10.046	1.141
pH	3	27.906	9.302	4.538	21.757	2.472
Temperature	3	790.709	263.569	128.592	784.56	86.148
Inoculum	3	6.309	2. 103	1.026	0.16	0.018
Other/Error	19	38.942	2.049			7.221

### Factor interaction, optimized conditions, and validation of enzyme production

Interactions among factors and levels of experiment can be analyzed by determining the severity index (SI), as shown in Table 4. The SI can be used to predict the effect of combined factors on enzyme production.

**Table 4.** Estimated interaction of severity index.

Interacting factor pairs	Columns	SI (%)	Col	Opt.
pH x Inoculum	1 × 2	69.72	3	2.1
L-asparagin x temperature	2 × 4	21.86	6	1.3
L-asparagin x Inoculum	1 × 4	14.84	5	2.3
L-asparagin x pH	1 × 3	13.7	2	2.2
temperature x Inoculum	3 × 4	13.38	7	2.3
pH x temperature	2 × 3	4.5	1	1.2

The SI measures the interaction between two factors. The analysis showed that the interaction between pH and inoculum was the highest with a SI of 69.72%, while the lowest SI of 4.5% was found for the interaction between pH and temperature. The interaction between pH and inoculum significantly influenced L-ASNase production. Conversely, the ANOVA results showed that the most influential factor for L-ASNase production was temperature. Different SI values could result in different values of individual factors ^21^.

### Optimum factors for enzyme production

L-ASN at a concentration of 2% (level 4), pH 6 (level 1), temperature 35°C (level 2), and an inoculum concentration of 1.5% (level 3) for 24 h of incubation were deduced as the best conditions for L-ASNase production with an expected enzyme production of 19.2065 U/ml ( Table 5).

**Table 5.** The predicted factors for optimum production of L-asparaginase.

Factor	Level description	Level	Contribution
L-asparagine	2%	4	0.556
pH	6	1	0.885
Temperature	35°C	2	8.127
Inoculum	1.5%	3	0.729
Total contribution from all factors			10.269
Current Grand Average of performance			8.909
Expected Result at Optimum condition			19.206

A validation experiment was required to confirm the expected result. Based on the confirmation test results, the L-ASNase activity obtained from *L. fusiformis* B27 at 24 h was 8.51 U/ml. The L-ASNase enzyme activity from *L. fusiformis* B27 at 24 h before and after optimization is shown in Table 6.

**Table 6.** Comparison of enzyme production.

Experiment	Enzyme production
**Control**	3.18 U/ml
**Expected result**	19.20 U/ml
**Validation result**	8.51 U/ml

The data showed that before optimization (control) L-ASNase had a low activity of 3.18 U/ml. This was because the bacterial environment for producing L-ASNase was suboptimal. The L-ASNase activity increased to 8.51 U/ml after optimization. The optimized factors for enzyme production could be predicted using the Taguchi method ^22– 24^. Confirmation experiment results are usually closer to the predicted values. However, these are sometimes lower than the predicted results. In this study, the optimized factors enhanced the enzyme production from 3.18 to 8.51 U/ml. This represented an almost three-fold increase. This increase was higher than that published in previous research ^21^ but lower than that in another study ^25^.

## Conclusions

*Lysinibacillus fusiformis* B27, which was isolated from mangrove, *Rhizophora mucronata,* can be optimized for L-ASNase enzyme production using optimization factors (L-ASNase, pH, temperature, and inoculum). Optimization using the Taguchi approach can increase L-ASNase enzyme production by approximately three-fold.

## Data availability

### Underlying data

Figshare: Raw data for production improvement of L-Asparaginase from *Lysinibacillus fusiformis*. https://doi.org/10.6084/m9.figshare.10265396.v2 ^26^

This project contains the following underlying data:

- L-ASNase activity for Taguchi analysis for 16 optimization experiments.
- Results for confirmatory experiment
- Images of all 31 isolates
- Uncropped, unedited blots from genome (B27)
- Sequence of B27
- Uncropped, unedited image of Gram stain of B27, as shown in Figure 1B

Data are available under the terms of the Creative Commons Attribution 4.0 International license (CC-BY 4.0).
