# Supplementary material for: Optimization of glutaminase-free L-asparaginase production using mangrove endophytic Lysinibacillus fusiformis B27
Source: F1000Res. 2020 May 14;8:1938. Originally published 2019 Nov 20. [Version 2] doi: 10.12688/f1000research.21178.2 (PMC7287513; doi:10.12688/f1000research.21178.2)
Supplement: Peer review history

# Peer review: Reviewer response for version 2

**DOI:** 10.5256/f1000research.26579.r63409

**Published:** 2020-06-10

**Authors:** Evans Manyara Nyakeri

**Affiliations:** 1 Africa Centre of Excellence in Sustainable Use of Edible Insects as Food and Feeds (INSEFOODS) and School of Agricultural and Food Sciences, Jaramogi Oginga Odinga University of Science and Technology, Bondo, Kenya

I have reviewed the corrections made by the authors against the suggestions I had made earlier and it is my conclusion that they have been addressed satisfactorily. I, therefore, approve the revised form of the manuscript.

Is the work clearly and accurately presented and does it cite the current literature?

Yes

If applicable, is the statistical analysis and its interpretation appropriate?

Yes

Are all the source data underlying the results available to ensure full reproducibility?

Partly

Is the study design appropriate and is the work technically sound?

Yes

Are the conclusions drawn adequately supported by the results?

Yes

Are sufficient details of methods and analysis provided to allow replication by others?

Partly

Reviewer Expertise:

Microbiology, biotechnology, food security and applied insect science.

I confirm that I have read this submission and believe that I have an appropriate level of expertise to confirm that it is of an acceptable scientific standard.

# Peer review: Reviewer response for version 2

**DOI:** 10.5256/f1000research.26579.r63408

**Published:** 2020-06-01

**Authors:** Sathyanarayana N. Gummadi

**Affiliations:** 1 Applied and Industrial Microbiology Lab, Department of Biotechnology, Indian Institute of Technology Madras, Chennai, India

The authors addressed all the queries except for the comment about the novelty:

 The values reported in this work are very low compared to commercial enzymes and other published reports. Authors claim that among endophytes this strain seems better. Authors need to justify in the introduction why L-asparaginases from endophytes are required when compared to E.coli and other bacterial and fungal strains.

Is the work clearly and accurately presented and does it cite the current literature?

Partly

If applicable, is the statistical analysis and its interpretation appropriate?

Partly

Are all the source data underlying the results available to ensure full reproducibility?

Partly

Is the study design appropriate and is the work technically sound?

No

Are the conclusions drawn adequately supported by the results?

Partly

Are sufficient details of methods and analysis provided to allow replication by others?

Yes

Reviewer Expertise:

NA

I confirm that I have read this submission and believe that I have an appropriate level of expertise to confirm that it is of an acceptable scientific standard.

# Peer review: Reviewer response for version 1

**DOI:** 10.5256/f1000research.23313.r62909

**Published:** 2020-05-07

**Authors:** Sathyanarayana N. Gummadi

**Affiliations:** 1 Applied and Industrial Microbiology Lab, Department of Biotechnology, Indian Institute of Technology Madras, Chennai, India

The study investigated the ability of mangrove *Rhizophora mucronata* to produce glutaminase-free L-asparaginase and then production enhancement by optimizing parameters L-asparagine concentration, pH, temperature, and inoculum concentration by Taguchi method.

 Numerous reports are available on glutaminase-free asparaginase producing microbial sources and considering this, results presented in this study are too preliminary and authors should think the novelty of this study.

 The following are the comments to improve the quality of the manuscript.

**Major comments:**

  

1. The Introduction part is looking very general like in many other published papers. There is no strong rationale to screen another strain that produced very low levels of L-asparaginase. 
2. It was mentioned in ASNase assay that 1.5 μl of Trichloro Acetic Acid 1.5 M was added to stop the reaction. Eager to know how this lower volume **1.5 μl** TCA will stop the reaction and can be accurate? It is better to include the equation to calculate the activity. 
3. The results of this study in terms of activity measured is very low (8.5 U/ml to 15 U/ml) compared to other commercial L-asparaginases ( *E. coli*) and other novel reported sources. Moreover, the study is titled the enzyme is ‘glutaminase free L-asparaginase’; however, no evident experiments or data reported in this study for claiming this.
4. Did the authors also check for urease activity?
5. Tables are numbered 1-5, showing only coding and interpretation results obtained from Taguchi statistical analysis. Many can be compiled as a single table and if necessary can be filed as supplementary data. For figure no.3, more description needed and clarify the 2 curves shown in each plot.
6. What is the rationale in choosing only four variables for optimization? Why did the authors screen the variables initially?
7.  Did the authors try to fit the experimental results to any quadratic equation?
8. How did the Taguchi experimental design optimize the four variables? How did authors arrive at the optimal values and verify?
9. At optimal settings, the expected result is ~ 19 U/ml but the authors obtained only 8.51 U/Ml. The huge variation between experimental and predicted values suggests that the design is not able to perform to good optimization.
10. There is no growth data provided in the manuscript. The experimental values represented in Table 1 corresponds to what time of growth?
11. The study showed mangrove *Rhizophora mucronata *as producers of L-asparaginase and optimization of its production parameters. More experiments must be included to reveal its biochemical properties, glutaminase free activity, molecular weight, and purity to explore it into application level as mentioned in the introduction part.

Is the work clearly and accurately presented and does it cite the current literature?

Partly

If applicable, is the statistical analysis and its interpretation appropriate?

Partly

Are all the source data underlying the results available to ensure full reproducibility?

Partly

Is the study design appropriate and is the work technically sound?

No

Are the conclusions drawn adequately supported by the results?

Partly

Are sufficient details of methods and analysis provided to allow replication by others?

Yes

Reviewer Expertise:

Industrial Biotechnology, Bioprocess Engineering,  Fermentation, Enzymology

I confirm that I have read this submission and believe that I have an appropriate level of expertise to confirm that it is of an acceptable scientific standard, however I have significant reservations, as outlined above.

# Response

**Published:** 2020-05-12

**Authors:** Asep Awaludin Prihanto

**Affiliations:** Faculty of Fisheries and marine Science, Brawijaya University, Indonesia

1. The Introduction part is looking very general like in many other published papers. There is no strong rationale to screen another strain that produced very low levels of L-asparaginase. 

**The reason for the need for exploring the new source is added to the first sentenced in the last paragraph.** 

2.  It was mentioned in ASNase assay that 1.5 μl of Trichloro Acetic Acid 1.5 M was added to stop the reaction. Eager to know how this lower volume **1.5 μl** TCA will stop the reaction and can be accurate? It is better to include the equation to calculate the activity. 

**We have miswritten the amount of the TCA, The TCA volume was revised.**

**Formula for calculating the enzyme activity was added to the manuscript**

3. The results of this study in terms of activity measured is very low (8.5 U/ml to 15 U/ml) compared to other commercial L-asparaginases ( *E. coli*) and other novel reported sources. Moreover, the study is titled the enzyme is ‘glutaminase free L-asparaginase’; however, no evident experiments or data reported in this study for claiming this.

**Several studies for endophytic bacteria showed lower production than these bacteria. For example the report from Hatamzadeh et al. (2020), PeerJ, DOI10.7717/peerj.8309.**

**The glutaminase free-L-asparaginase activity was confirmed in our preliminary experiment when investigated the activity onto M9 modified agar supplemented L-Asparagine and L-Glutamine. In the L-Glutamine –supplemented M9 media, the bacteria don’t show the activity (the color of media remains yellow). Meanwhile, Asparagine –supplemented M9 media turn blue. We also confirmed the nil activity of the cell-free supernatant**.

4. Did the authors also check for urease activity?

**No, we did not check the Urease activity**

5. Tables are numbered 1-5, showing only coding and interpretation results obtained from Taguchi statistical analysis. Many can be compiled as a single table and if necessary can be filed as supplementary data. For figure no.3, more description needed and clarify the 2 curves shown in each plot.

**We afraid if we compiled the Tables, then the clarity and the flowing of the manuscript will also difficult to be followed. Hence, we prefer to show the detail on the tables as in the manuscript. The additional description of the manuscript was added.**

6. What is the rationale in choosing only four variables for optimization? Why did the authors screen the variables initially?

**We have performed the initial investigation toward five individual factors namely, temperature, L-ASN, pH, inoculum size, and shaker speed. In our preliminary investigation, shaker speed failed to give a significant effect on the production of L-ASNase. Hence, we only applied four variables (L-ASN, temp, pH, and inoculum size).**

****

7. Did the authors try to fit the experimental results to any quadratic equation?

**No, we did not, in the Taguchi method we automatically are recommended the best equation /formula in order to obtain the best production**.

8. How did the Taguchi experimental design optimize the four variables? How did authors arrive at the optimal values and verify?

**By using Qualitek-4 software for Taguchi experimental, we directly we are automatically directed with the formulation for the production of the most optimum enzymes accompanied by an estimate of its production (Table 5.)**

9. At optimal settings, the expected result is ~ 19 U/ml but the authors obtained only 8.51 U/Ml. The huge variation between experimental and predicted values suggests that the design is not able to perform to good optimization.

**It is true that the result of the estimated production is higher than that of the exact production. However many researchers also experienced a similar result, where the exact value is lower than the predicted one. As examples is reported by** **Chenthamarakshan, A., Parambayil, N., Miziriya, N. *et al.* Optimization of laccase production from *Marasmiellus palmivorus* LA1 by Taguchi method of Design of experiments. *BMC Biotechnol* 17, 12 (2017). https://doi.org/10.1186/s12896-017-0333-x; and Shehata AN,  Abd El Aty AA. 2014. Optimization of Process Parameters by Statistical Experimental Designs for the Production of Naringinase Enzyme by Marine Fungi. International Journal of Chemical Engineering. ID 273523. https://doi.org/10.1155/2014/273523. The main focus is on the increase in production by comparing before and after optimization.**

**In the optimized formulation, we reached the increase of the production by three folds. Hence, we believe that the optimization worked.**

10. There is no growth data provided in the manuscript. The experimental values represented in Table 1 corresponds to what time of growth?

**The culture time is 24 h. we have revised the manuscript to clarify the time of harvesting**

11. The study showed mangrove *Rhizophora mucronata *as producers of L-asparaginase and optimization of its production parameters. More experiments must be included to reveal its biochemical properties, glutaminase free activity, molecular weight, and purity to explore it into application level as mentioned in the introduction part.

**We have confirmed the glutaminase free activity by investigating using L-glutamine as a substrate. The further characteristic has been under investigation. Next paper will discuss the purification and characteristic of the enzyme from *Lysinibacillus fusiformis* B27**

****

# Peer review: Reviewer response for version 1

**DOI:** 10.5256/f1000research.23313.r56818

**Published:** 2019-12-18

**Authors:** Evans Manyara Nyakeri

**Affiliations:** 1 Africa Centre of Excellence in Sustainable Use of Edible Insects as Food and Feeds (INSEFOODS) and School of Agricultural and Food Sciences, Jaramogi Oginga Odinga University of Science and Technology, Bondo, Kenya

The study topic is important as it clearly illustrates the need for optimising the production of the enzyme L-ASNASE. 

 I however have a few suggestions that can contribute towards improving the work:

1. The authors should consider revision of the topic to capture the gist of the study which isolation endophytic bacteria that produce the enzyme glutaminase free L-ASNASE mangrove forest. Suggestion: Isolation of L-ASNASE producing endophytic bacteria and optimization of production of the enzyme. The topic as it is suggest that the study was targeting the isolation of the *Lysinibacillus fusiformis* B27 yet this was the finding of the study rather than the intentional target as from the start. 
2. The study is not clearly bringing out the problem of the study: For example: the study rightly points out the limitations of L-ASNAS in paragagh 2 and 3. However, it does not point out the source of L-ASNAS with these limitations and the reason for advocating for alternative sources such as the endophytic bacteria from the mangrooves. Furthermore, the reason given for advocating the sourcing from microbes is not consistent in solving the prior identified limitations of the enzyme: the rapid multiplication and easy management of microbes vis avis the sensitivity to high temperatures (ie. need for extremophilic enzyme). 
3. The study has not clearly stated the relationship between substrate glutamine and the enzyme L-ASNase and the need for microbes able to produce asparaginase and not glutaminase nor both. This needs to be established in the introduction. Is it because the two enzymes are antagonistic? The idea was to screen for bacteria isolates capable of producing only the enzyme asparaginase and not glutaminase or both gluataminase and asparaginase., Why discriminate on glutaminase? What is the basis for this? This has not been explained. Is is because the two enzymes are antagonistic? 
4. Again, how did you identify isolates that were producing the enzymes? Formation of clearance zones, testing of media for presence of substrates and substrate level reduction, change of media colour especially due to bromothymol blue indicator? Explain to make this clearer.
5. The methodology on screening of enzyme producing bacterial isolates is not conclusively described. Suggestion: state that the plating of the bacteria on the two media types was done by replica plating.
6. The molecular study is adequate in methodology, results and interpretation. 

Is the work clearly and accurately presented and does it cite the current literature?

Yes

If applicable, is the statistical analysis and its interpretation appropriate?

Yes

Are all the source data underlying the results available to ensure full reproducibility?

Partly

Is the study design appropriate and is the work technically sound?

Yes

Are the conclusions drawn adequately supported by the results?

Yes

Are sufficient details of methods and analysis provided to allow replication by others?

Partly

Reviewer Expertise:

Microbiology, biotechnology, food security and applied insect science.

I confirm that I have read this submission and believe that I have an appropriate level of expertise to confirm that it is of an acceptable scientific standard, however I have significant reservations, as outlined above.

# Response

**Published:** 2019-12-23

**Authors:** Asep Awaludin Prihanto

**Affiliations:** Faculty of Fisheries and marine Science, Brawijaya University, Indonesia

I, however, have a few suggestions that can contribute towards improving the work:

1. The authors should consider revision of the topic to capture the gist of the study which isolation endophytic bacteria that produce the enzyme glutaminase free L-ASNASE mangrove forest. Suggestion: Isolation of L-ASNASE producing endophytic bacteria and optimization of production of the enzyme. The topic as it is suggest that the study was targeting the isolation of the *Lysinibacillus fusiformis* B27 yet this was the finding of the study rather than the intentional target as from the start. 

-->As suggested by the reviewer, we revised the title to “ **Isolation of glutaminase-free L-asparaginase  producing endophytic bacteria and optimization of production of the enzyme**”

2. The study is not clearly bringing out the problem of the study: For example: the study rightly points out the limitations of L-ASNAS in paragraphs 2 and 3. However, it does not point out the source of L-ASNAS with these limitations and the reason for advocating for alternative sources such as the endophytic bacteria from the mangroves. Furthermore, the reason given for advocating the sourcing from microbes is not consistent in solving the prior identified limitations of the enzyme: the rapid multiplication and easy management of microbes vis avis the sensitivity to high temperatures (ie. need for extremophilic enzyme). 

-->We added one sentence before the end of paragraph 3 with “ **…….The drawbacks of the enzyme applications might be overcome by isolating the enzyme from a unique habitats, such as an endophytic bacteria from mangrove.** **Therefore, new L-ASNase producers need to be explored** **. ** 

3. The study has not clearly stated the relationship between substrate glutamine and the enzyme L-ASNase and the need for microbes able to produce asparaginase and not glutaminase nor both. This needs to be established in the introduction. Is it because the two enzymes are antagonistic? The idea was to screen for bacterial isolates capable of producing only the enzyme asparaginase and not glutaminase or both gluataminase and asparaginase., Why discriminate on glutaminase? What is the basis for this? This has not been explained. It is because the two enzymes are antagonistic? 

-->We have revised the third sentence on paragraph 3 with” **….** **In medical applications, it has a low half-life, allergic reactions, and unnecessary glutamine hydrolysis on L-asparaginase due to the lack of** ***asparagine synthetase*** **in cancer cells.**

4. Again, how did you identify isolates that were producing the enzymes? Formation of clearance zones, testing of media for the presence of substrates and substrate-level reduction, change of media colour especially due to bromothymol blue indicator? Explain to make this clearer.

-->We have revised the paragraph on Section of Result and Discussion, subsection Screening and Identification with “ **The screening results showed that 31 endophytic mangrove isolates could produce the L-ASNase enzyme shown by blue color-formed surrounding colony (** **Figure 1A). The change of the color due to the increase of pH resulting from enzyme activity. The best enzyme producer was isolate B27. This isolate had a more extensive and darker blue tone compared to other isolates. Furthermore, only isolate B27 produced glutaminase free-L-asparaginase. Hence, further molecular identification was applied only to the B27 isolate. Further, Gram stain analysis revealed that the bacterium was Gram-positive ( Figure 1B)**.

5. The methodology on screening of enzyme-producing bacterial isolates is not conclusively described. Suggestion: state that the plating of the bacteria on the two media types was done by replica plating.

-->We have revised on Section Methods, subsection Isolation and screening of L-ASNase producers in paragraph two, sentence six with “All isolates were streaked onto two different media (M9 medium with L-ASN, and M9 medium with L-glutamine) in duplicates. The plates were incubated at 37°C overnight.  

6. The molecular study is adequate in methodology, results and interpretation. 

-->Thank you for your comment
